# Complex Relationship between Mismatch Repair Proteins and MBD4 during Immunoglobulin Class Switch Recombination

**DOI:** 10.1371/journal.pone.0078370

**Published:** 2013-10-29

**Authors:** Fernando Grigera, Alfonso Bellacosa, Amy L. Kenter

**Affiliations:** 1 Department of Microbiology and Immunology, University of Illinois College of Medicine, Chicago, Illinois, United States of America; 2 Cancer Epigenetics and Cancer Biology Programs, Fox Chase Cancer Center, Philadelphia, Pennsylvania, United States of America; Université de Montréal, Canada

## Abstract

Mismatch repair (MMR) safeguards against genomic instability and is required for efficient Ig class switch recombination (CSR). Methyl CpG binding domain protein 4 (MBD4) binds to MutL homologue 1 (MLH1) and controls the post-transcriptional level of several MMR proteins, including MutS homologue 2 (MSH2). We show that in WT B cells activated for CSR, MBD4 is induced and interacts with MMR proteins, thereby implying a role for MBD4 in CSR. However, CSR is in the normal range in Mbd4 deficient mice deleted for exons 2–5 despite concomitant reduction of MSH2. We show by comparison in Msh2^+/−^ B cells that a two-fold reduction of MSH2 and MBD4 proteins is correlated with impaired CSR. It is therefore surprising that CSR occurs at normal frequencies in the Mbd4 deficient B cells where MSH2 is reduced. We find that a variant Mbd4 transcript spanning exons 1,6–8 is expressed in Mbd4 deficient B cells. This transcript can be ectopically expressed and produces a truncated MBD4 peptide. Thus, the 3′ end of the Mbd4 locus is not silent in Mbd4 deficient B cells and may contribute to CSR. Our findings highlight a complex relationship between MBD4 and MMR proteins in B cells and a potential reconsideration of their role in CSR.

## Introduction

In mature B cells activation induced cytidine deaminase (AID) initiates immunoglobulin (Ig) class switch recombination (CSR), an event that diversifies Ig effector function by swapping the constant (C) region associated with the V(D)J exons ( [1] and references therein). During CSR two distal DNA switch (S) regions, each paired with C_H_ regions, come into close proximity and undergo reciprocal recombination [2]. CSR causes the looping out and deletion of the DNA between two S regions targeted for recombination resulting in a rearrangement of the *Igh* locus. CSR culminates with switching from IgM to one of six secondary isotypes (in mouse, IgG3, IgG1, IgG2b, IgG2a, IgE, IgA). CSR critically depends on the formation of double-stranded breaks (DSBs). The sequential actions of AID, uracil DNA glycosylase (UNG), and apurinic/apyrimidinic endonucleases 1 and 2 (APE1 and APE2) create single-stranded breaks (SSBs) that result in a DSB if two SSBs are close and on opposing strands in S DNA. However, when SSBs are distant, mismatch repair (MMR) proteins are required to effectively process SSBs into DSBs [3]. Thus, efficient initiation of CSR leading to the formation of DSBs in S regions requires AID, BER and MMR processes. Completion of the CSR reaction requires that DSBs are resolved by nonhomologous end joining DNA repair [4].

We focus here on MBD4 because it has several features that make it an attractive candidate for a role in CSR. Like UNG, MBD4 possesses strong U/G mismatch DNA glycosylase activity [5]. MBD4 is distinct from UNG in that it also interacts with and stabilizes MLH1, an MMR protein [6], [7]. The interaction between MLH1 and MBD4 has been postulated to play a role in the coordination of BER and MMR to rectify T:G and U:G mismatches [8]. However, targeted deletion of Mbd4 exon three in mice does not impair CSR in B cells [9]. This is a surprising result because in mouse embryonic fibroblasts (MEFs) MBD4 stabilizes MMR proteins [6], which are required for CSR. However, it is not clear what the minimum MMR protein concentration requirement is for WT levels of CSR.

We have examined the role of Mbd4 in isotype switching and MMR protein stability. Co-IP studies indicate that endogenous MBD4 interacts with the MMR proteins, MLH1 and postmeiotic segregation increased 2 (PMS2) in activated B cells. Using Mbd4 deficient mice in which exons 2–5 were removed by targeted deletion [6] and referred to here as Mbd4^Δ2−5/Δ2−5^, we found that in activated B cells MLH1 and MSH2 levels are indeed reduced, whereas CSR is not impaired. In contrast, Msh2^+/−^ B cells were haploinsufficient for IgG3 and IgG1 switching and both MSH2 and MBD4 proteins were reduced. It was surprising that Mbd4^Δ2−5/Δ2−5^ B cells support WT levels of CSR since MSH2 proteins levels are diminished below those found in Msh2^+/−^ B cells. Therefore, we examined Mbd4^Δ2−5/Δ2−5^ B cells for residual Mbd4 expression that might explain retention of CSR function. In activated Mbd4^Δ2−5/Δ2−5^ B cells we detected a variant Mbd4 transcript spanning exons 1,6–8 and which upon ectopic expression produces a truncated MBD4 peptide in CH12.F3 cells. Our studies highlight a complex relationship between MBD4, and MMR proteins and suggest that reconsideration of the role for Msh2 in CSR may be warranted.

## Results

### MBD4 is induced in activated B cells and interacts with MLH1 and PMS2

**Figure 1:**
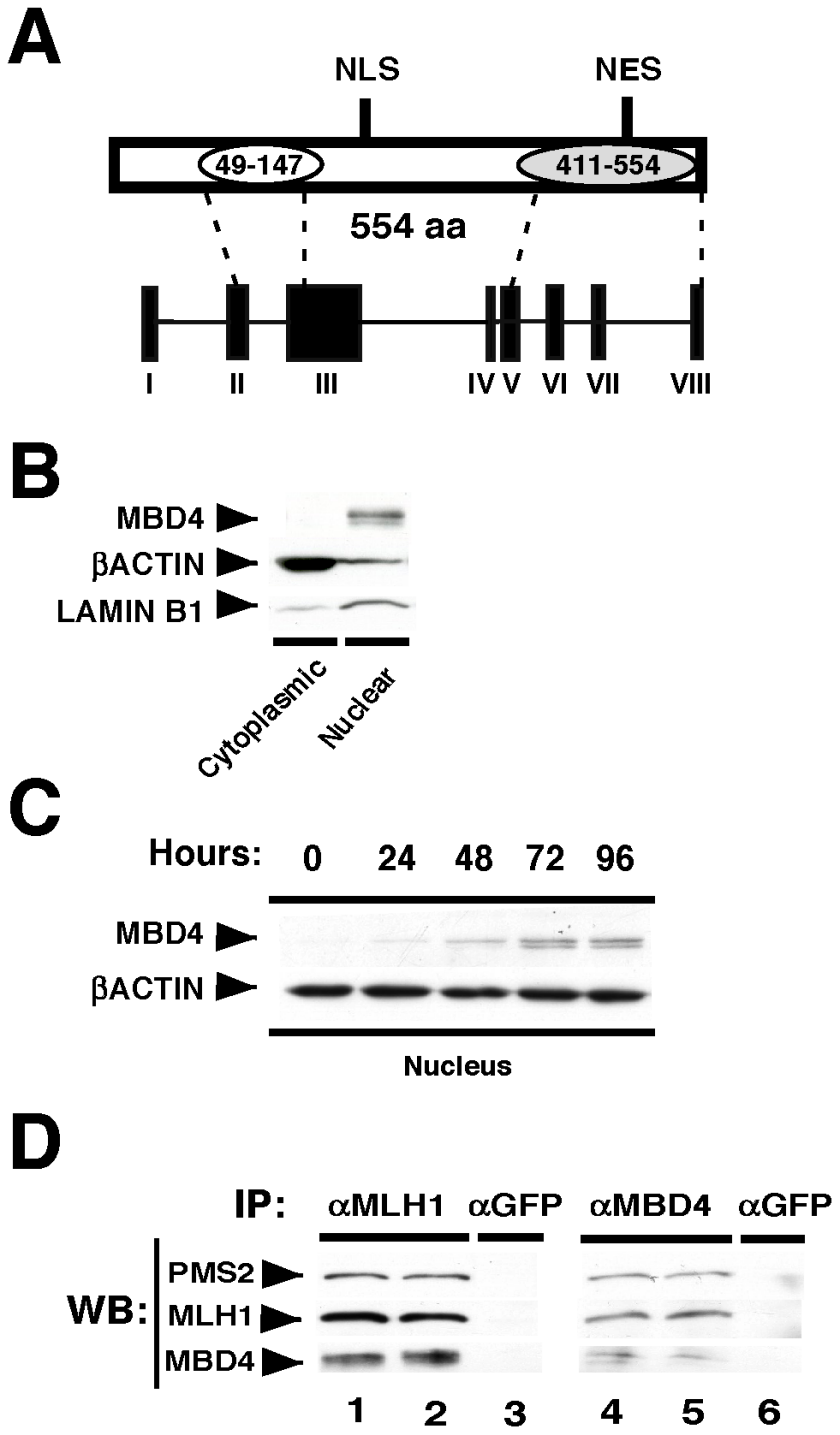
MBD4 is induced in activated B cells and interacts with MLH1 and PMS2. **A)** A schematic of the murine MBD4 protein, including the methyl-binding domain (MBD) (white oval; aa 49–147), the glycosylase domain (gray oval; aa 411–554) spanning exons II–III and V–VIII, respectively, and predicted NLS and NES. **B,C**) Resting splenic B cells were activated with LPS+IL4 for (**B**) 72 hours or (**C**) the indicated times. **B**) Total protein was determined by Bradford assay and either cytoplasmic (100 µg), or nuclear (25 µg) extracts were examined in a Western blot with Abs against MBD4, β-ACTIN, and LAMIN B1. **C)** Nuclear (50 µg) extracts were examined in Western analysis using anti-MBD4 and anti-β-ACTIN Abs. Extracts were prepared from two independent mice. One representative experiment is shown. **D**) Nuclear extracts from CH12.F3 cells induced with CIT for 24 hours were immunoprecipitated with α-MLH1, α-MBD4, or α-GFP Abs then analyzed by Western blot (WB) with the indicated Abs. IPs were performed in duplicate in two independent experiments. One representative experiment is shown.

Murine MBD4 contains a methyl-binding domain and a glycosylase domain, encoded by exons 2–3 and 5-8, respectively (fig. 1A) [10], [11]. To begin we examined MBD4 expression in resting splenic B cells that were activated with LPS+IL4 then harvested at various time points and analyzed by Western blot. MBD4 expression is evident by 72 hours of B cell stimulation but is absent in the cytoplasm (fig. 1B,C). To confirm the purity of the nuclear and cytoplasmic extracts we tested both anti-LAMINB1 and anti-βACTIN antibodies (Abs) in Western analyses on samples normalized by quantitative Bradford assay. LAMINB1 is clearly enriched in the nuclear extract and depleted in the cytoplasmic extract, whereas βACTIN displays a reciprocal enrichment pattern, which confirms the efficacy of our extract preparations (fig. 1B). LAMIN B1 co-migrates with MBD4, making it difficult to visualize both proteins on the same membrane. To circumvent this issue we use βACTIN as a nuclear loading control throughout this report, since 1) studies have shown that βACTIN is present in the nucleus [12], [13], [14] and 2) we find samples handled under identical conditions and normalized by quantitative Bradford show equivalent amounts of βACTIN in Western analysis (data not shown). Thus in resting B cells activated with LPS+IL4 we detect expression of two MBD4 isoforms specifically in nuclei that increases over time compared to the loading control (fig. 1C). The slower migrating isoform begins expression at 24 hours, while the faster isoform appears later, at approximately 48 hours (fig. 1B). To better appreciate MBD4 nuclear localization, we searched for the presence of nuclear localization signal (NLS) and nuclear export signal (NES) that might explain its preferential compartmentalization in B cells. We found a predicted NLS (aa 214-KRRKSKR-220) in exon 3 [15], [16] and a predicted NES (aa 482-LGLYDL-487) in exon 7 [17] suggesting that nuclear localization may require active nuclear import (fig. 1A) [15].

MBD4 was identified as a MLH1 interaction-partner in yeast two-hybrid screens [7] and MMR proteins are actively localized to the nucleus [18], [19]. We examined the capability of MBD4 to interact with MLH1 in the CH12.F3 cell line that undergoes inducible CSR when activated with CD40L+IL4+TGFβ (CIT) [20]. Nuclear extracts were incubated with anti-MLH1, or anti-GFP control Abs, and then examined for co-IP partners by Western. First, we tested for the integrity of the MLH1:PMS2 complex [21] and the capability of MLH1 to interact with MBD4. IP with anti-MLH1 pulls down MLH1 and PMS2, as well as MBD4 (fig. 1D, lane 1–2). In contrast, the anti-GFP control does not IP any of these proteins (fig. 1D, lane 3). Reciprocally, IP with anti-MBD4 pulls down MBD4, MLH1 and PMS2 whereas these proteins are absent when anti-GFP is used (fig. 1D, lanes 4–6). We conclude that MLH1 and MBD4 interact with each other and with PMS2 in activated CH12.F3 cells. These findings raise the possibility that MBD4 contributes to CSR through its interaction with MMR proteins.

### MMR protein stability is dependent on intact MBD4 in activated B cells

**Figure 2:**
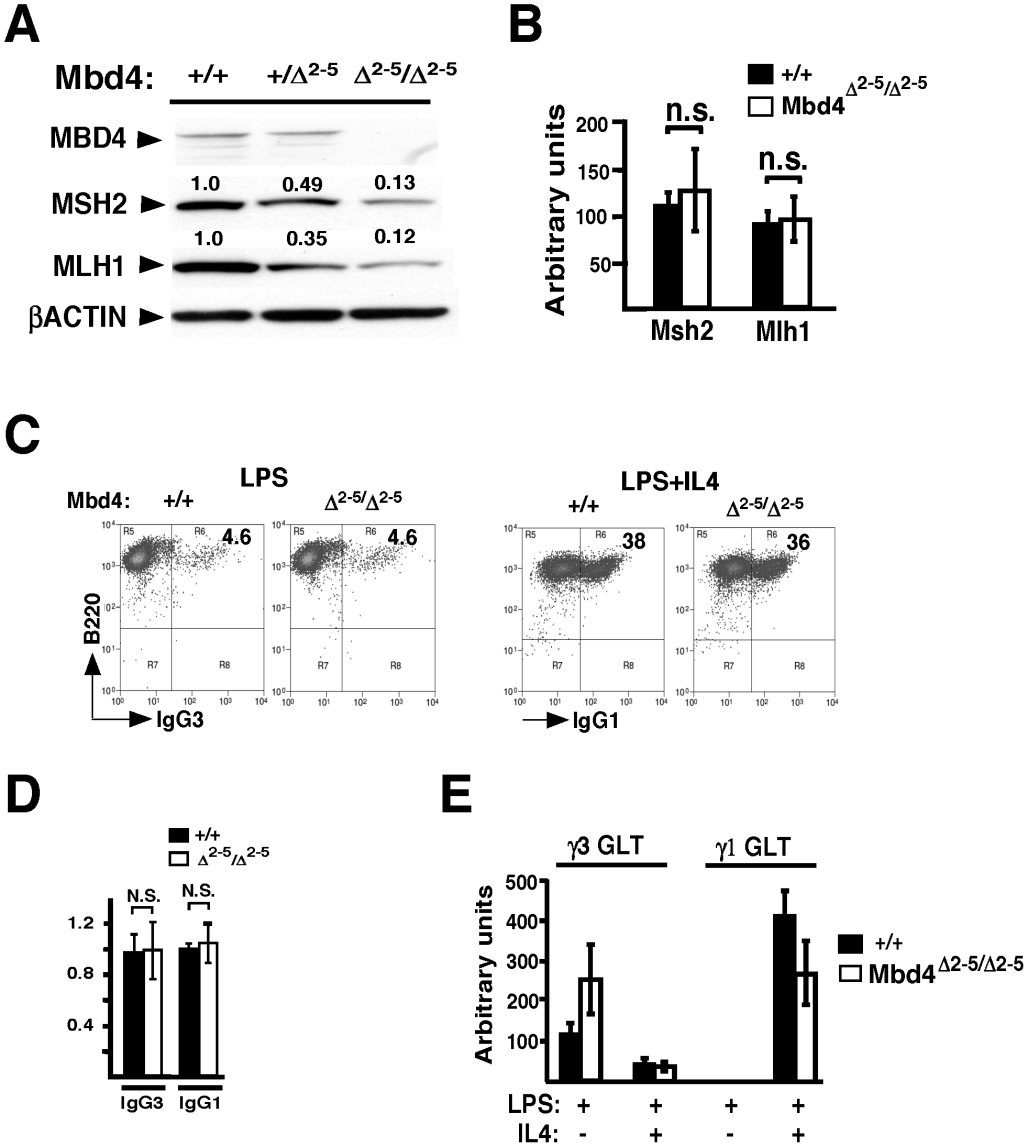
Mbd4 deficient B cells undergo CSR despite reduced MSH2 and MLH1. Splenic B cells from WT, Mbd4^Δ2−5/+^, or Mbd4^Δ2−5/Δ2−5^ mice were activated with LPS+IL4 for 48 hours and nuclear extracts (**A**) or cDNA (**B**) were prepared in two independent experiments. **A**) Immunoblot analyses of nuclear extracts were developed with Abs against MBD4 (69 kD), MSH2 (107 kD), MLH1 (95 kD), and β-ACTIN (43 kD). One representative experiment is shown. **B**) Mlh1 or Msh2 transcripts were analyzed by qRT-PCR and normalized to 18S rRNA. The averages from 2 to 3 independent experiments are shown with standard error of mean (SEM). **C-E**) Splenic B cells from WT or Mbd4^Δ2−5/Δ2−5^ from two independent mice were activated with LPS or LPS+IL4 to induce IgG3 or IgG1 CSR, respectively, for 4 days (**C,D**), or 48 hours (**E**). **C**) A representative FACS analysis of anti-B220 (APC) versus anti-IgG1 or anti-IgG3 (FITC) is shown. **D**) Cumulative CSR frequencies are shown with SEM. WT was set to 1. **E**) GLT γ3 and γ1 expression were analyzed by qRT-PCR and normalized to 18S rRNA and SEM are shown.

MMR heterodimers MSH2/MSH6 and MLH1/PMS2 are required for efficient CSR [3]. Mbd4 deficiency leads to reduced MMR proteins levels in MEFs, and this reduction is post-transcriptional [6]. Because MSH2 and MSH6 deficiencies create identical CSR deficits [22] and PMS2 stability is dependent on the MLH1 C-terminus [23] we focused on analyzing MLH1 and MSH2 in activated B cells to determine whether MMR protein levels and CSR are impaired in Mbd4 deficient B cells. In B cells activated with LPS+IL4, MSH2 and MLH1 were diminished by 87% and 88%, respectively, in Mbd4 deficient nuclei relative to WT (fig. 2A). In B cells, MMR protein levels are determined by post-transcriptional events because Msh2 and Mlh1 transcript levels are unaffected by Mbd4 deficiency (fig 2B). However, it is unclear whether CSR requires MBD4.

Since deletion of various MMR genes impairs CSR [24], [25], we sought to determine whether Mbd4^Δ2−5/Δ2−5^ deficiency impacts on switching efficiency. LPS and LPS+IL4 induce IgG3 and IgG1 switching respectively [2]. WT or Mbd4 deficient B cells were activated with LPS or LPS+IL4 for 72 hours and analyzed by FACS. Surprisingly, in two independent analyses, Mbd4^Δ2−5/Δ2−5^ B cells display no impairment of CSR or γ1 and γ3 germline transcript (GLT) expression (fig. 2C-E). However, since MSH2 and MLH1 protein levels were significantly diminished in activated Mbd4^Δ2−5/Δ2−5^ B cells, these findings indicate that very low levels of MMR proteins may be sufficient for robust CSR.

### Msh2 but not Mlh1 is haploinsufficient for CSR

**Figure 3:**
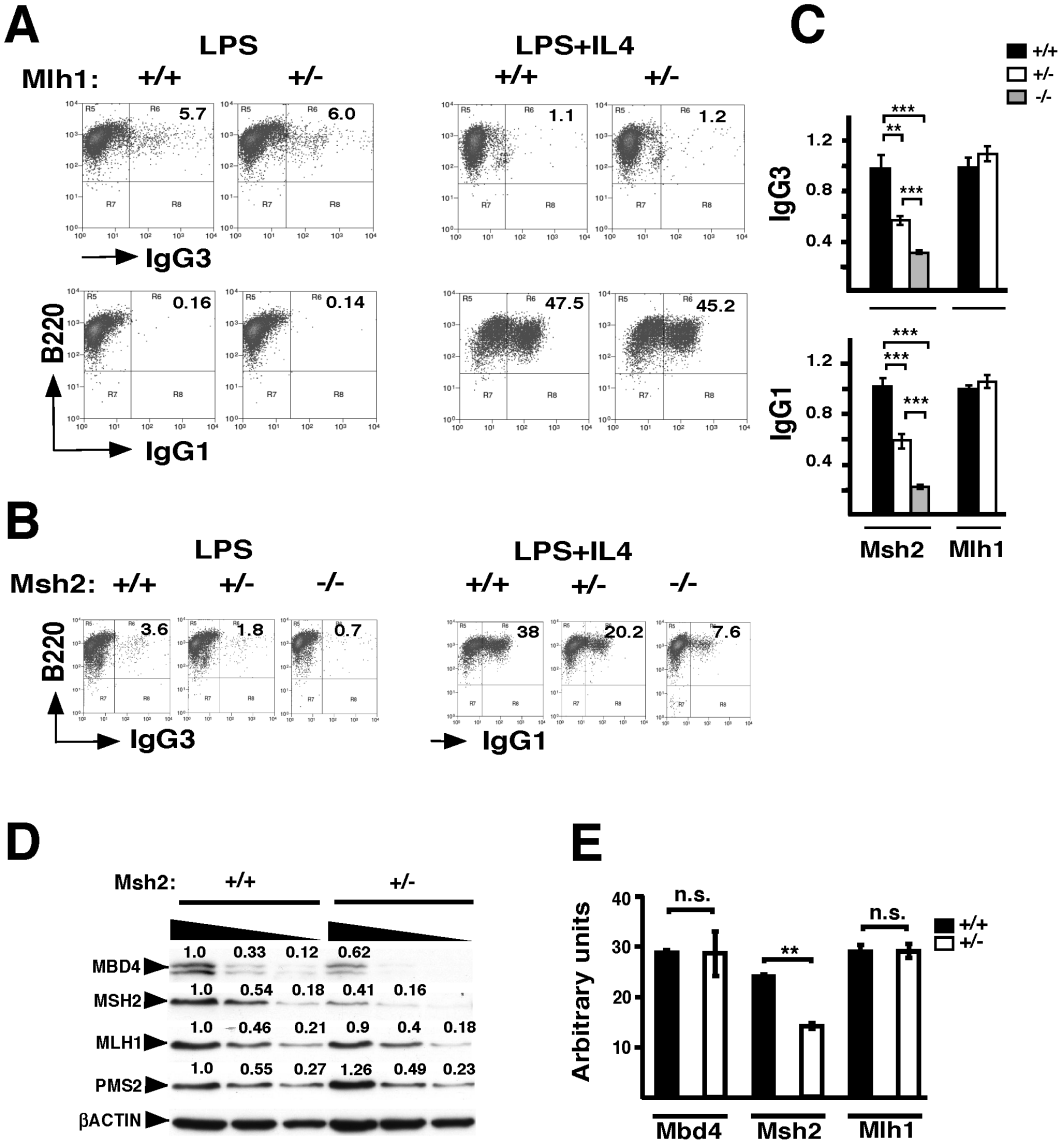
Msh2 ^+/^^−^ B cells are happloinsufficient for CSR. Splenic B cells from WT and Mlh1^+/−^ (**A**) or WT and Msh2^+/−^ (**B**) littermates were stimulated with LPS or LPS+IL4 to induce IgG3 or IgG1 switching, respectively. P values are from Student’s two tailed *t* test; p <0.05 (*), p<0.01 (**), p<0.001 (***). **A, B**) Two-three independent mice from each genotype were analyzed. Representative FACS analyses of anti-B220 (APC) versus anti-IgG1 or anti-IgG3 (FITC) are shown for WT, Mlh1 (**A**) and Msh2 (**B**) deficient B cells. **C**) CSR frequency averages from FACS experiments are shown with SEM. WT was set to 1. **D, E**) Splenic B cells from two independent WT and Msh2^+/−^ mice were activated with LPS+IL4 for 72 hours (**D**), or 48 hours (**E**). **D**) Nuclear extracts were analyzed by immunoblot for MMR protein expression, as indicated. Proteins were quantitated by densitometry and normalized to the βACTIN loading control. WT is set to 1. Triangles represent serial two fold dilutions of nuclear extracts. **E**) Averages of Q-RT-PCR of Msh2, Mbd4, and Mlh1 transcripts are normalized to 18S rRNA and shown with SEM.

We took two complementary approaches to establish the effect of MMR gene dosage on CSR. First, we tested LPS or LPS+IL4 activated B cells from WT and Mlh1^+/−^ or WT, Msh2^+/−^ and Msh2^−/−^ littermates for CSR frequency. In LPS induced WT B cells IgG3 but not IgG1 switching was found in 5.7% of B cells, whereas LPS+IL4 stimulated IgG1 but not IgG3 switching in 47.5% of B cells (fig. 3A). The IgG3 and IgG1 analyses for B cells activated with LPS+IL4 or LPS alone, respectively, are shown as controls for CSR specificity by these inducers. We found that IgG1 and IgG3 switching frequencies for Mlh1^+/−^ B cells are identical to WT littermate controls demonstrating that MLH1 protein is not limiting (fig. 3A). In contrast, CSR is reduced approximately 2-fold in Msh2^+/−^ B cells relative to WT, while Msh2^−/−^ B cells are reduced about 5-fold, demonstrating that Msh2 is haploinsufficient for both IgG3 and IgG1 switching (fig. 3B). Cumulative switching analyses confirm that CSR is significantly reduced in Msh2^+/−^ and Msh2^−/−^ B cells relative to WT, but not for Mlh1 ^+/−^ or Mbd4^Δ2−5/Δ2−5^ B cells (fig. 2D, 3C).

Next, we sought to determine whether reduced MSH2 protein levels destabilize MBD4 or other MMR proteins. Following LPS+IL4 activation, we quantitatively analyzed MBD4 and MMR protein and transcript levels in WT and Msh2^+/−^ B cells. Analyses indicate that levels of MSH2 protein and gene transcripts in Msh2^+/−^ B cells are about half that detected in WT B cells as assessed by Western and qRT-PCR, respectively (fig. 3D,E). We found that the MLH1 protein and transcript levels are unperturbed in Msh2^+/−^ B cells (fig. 3D,E). Similarly, PMS2 stability, which is dependent on the integrity of the MLH1 C-terminus [23], is also intact in Msh2^+/−^ B cells (fig. 3D). In contrast, in three independent experiments we found a reduction of MBD4 protein in Msh2^+/−^ B cells, whereas Mbd4 transcript levels remain unchanged (fig. 3D,E). Together these findings indicate that even a 2-fold reduction of MSH2 is sufficient to impair CSR in Msh2^+/−^ B cells and alter MBD4 stability. The co-reduction of MSH2 and MBD4 in Msh2^+/−^ B cells raises a question regarding the protein(s) that is actually required for CSR. If a 2-fold reduction of MSH2 proteins levels in Msh2^+/−^ B cells was sufficient to diminish CSR, then what accounts for the apparently intact CSR frequencies in the Mbd4 deficient B cells in which MSH2 levels are reduced?

### A truncated Mbd4 transcript is detected in Mbd4^Δ2−5/Δ2−5^ B cells activated for CSR

**Figure 4:**
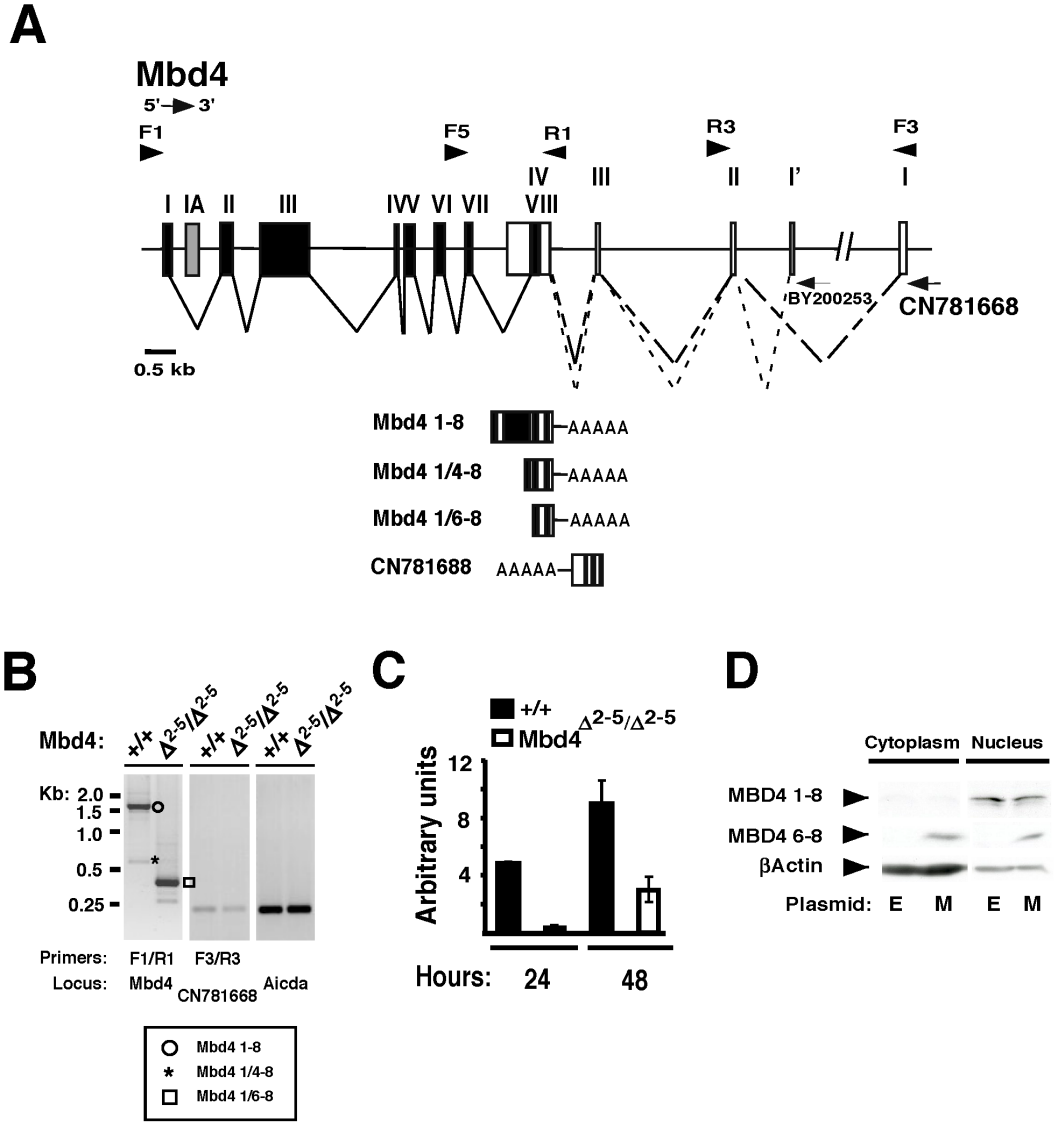
Exon 6 Kozak sequence directs expression of a truncated MBD4 peptide. **A**) Diagram of the Mbd4 locus, (chr6: 115,840,698-115,853,371 (mm8)) with a partial representation of the antisense CN781668 and BY200253 lncRNAs. The RNA splicing pattern for the full length Mbd4 transcript (solid line) is shown with exons 1–8 depicted as black blocks. The splicing patterns for CN781668 exons 1–4 (long dashed lines) and BY200253 (short dashed lines) are shown as white blocks. PCR primers (arrowheads) are indicated. **B,C**) WT or Mbd4^Δ2−5/Δ2−5^ B cells were activated with LPS+IL4 for 48 hours (**B**), or as indicated (**C**) and cDNA prepared. **B**) RT-PCR was carried out for Mbd4 (primers F1/R1), lncRNA CN781668 (primers F3/R3), or AID. Mbd4 1–8 (circle), Mbd4 1/4–8 (asterisk), Mbd4 1/6–8 (square) are indicated. In the lower panel a schematic is shown of Mbd4 and CN781668 transcripts with exons indicated by alternating black and white blocks followed by poly-A tails. **C**) QRT-PCR using F5/R1 primers detect WT Mbd4 1-8 and Mbd4^Δ2−5/Δ2−5^ 1/6–8 transcripts. Results are averaged from 2 independent experiments and shown with SEM. **D**) CH12.F3 cells were transduced in three independent experiments with empty (E) or Mbd4 exons 6–8 (M) expression constructs. Transductants were stimulated with CIT for 24 hours, nuclear extracts prepared and then analyzed by Western with anti-MBD4 or anti-βACTIN and a representative blot is shown.

The Mbd4 locus includes eight exons, an alternative exon 1A, and partially overlaps with the long non-coding RNAs (lncRNA), CN781668 and BY200253, expressed in the antisense direction (fig. 4A). We find that using the F3/R3 primer pair in RT-PCR lncRNA expression is detected in both WT and Mbd4^Δ2−5/Δ2−5^ B cells activated with LPS+IL4, as compared to the control, AID (fig. 4B). EST expression patterns indicate that alternative transcripts can be produced from the Mbd4 locus (AceView Gene Models with Alternative Splicing; Alternative Splicing Graph from Swiss Institute of Bioinformatics, NC_000072_2104). In PCR assays using primers F1/R1, we amplified full-length Mbd4 exons 1–8, as well as a minor species of about 0.5 kb from WT LPS+IL4 activated B cells (fig. 4B). DNA sequence analyses confirmed the identity of the 1.5 kb moiety as the canonical Mbd4 transcript and identified the 0.5 kb fragment as a variant beginning at exon 1, skipping to exon 4 and continuing through exon 8 (Mbd4 1/4–8) (fig. 4B). These findings confirm that alternative transcripts are detectable even in the WT context. In Mbd4^Δ2−5/Δ2−5^ B cells, where the full length Mbd4 transcript is absent, we found a truncated transcript spanning approximately 0.3 kb (fig. 4B). DNA sequence analysis revealed that the aberrant transcript initiates in exon 1, skips to exon 6 and then continues through exon 8 (Mbd4 1/6–8) (fig. 4B). A similar truncated Mbd4 transcript has been reported for the Mbd4 exon 3 knockout mouse that is also intact for CSR [9], [26]. Analysis of Mbd4 expression in human cells revealed both full length and short form transcripts [27]. The human Mbd4 short form lacked the methyl binding domain and exon 3 but retained the glycosylase domain, which possesses uracil excision activity [27]. Hence the propensity of Mbd4 to express alternatively spliced transcripts is not unique to the mouse or to genetically manipulated loci.

We compared the expression profile of the full length and Mbd4 1/6–8 transcripts from WT and Mbd4^Δ2−5/Δ2−5^ B cells, respectively. B cells were resting or activated with LPS+IL4 for 24 or 48 hours and monitored for Mbd4 transcripts using primers F5/R1 in qRT-PCR assays (fig. 4A). WT Mbd4 expression increases between 24 and 48 hours in accord with the Western analysis of MBD4 protein levels (fig. 1B,C, 4C). Mbd4 1/6–8 from Mbd4 deficient cells achieves an expression level equivalent to one-third that found for full length Mbd4 in WT at 48 hours of B cell activation (fig. 4C). Therefore we asked could Mbd4 1/6–8 produce a truncated peptide in Mbd4^Δ2−5/Δ2−5^ B cells?

### Exon 6 Kozak sequence supports ectopic expression of truncated MBD4

In the endogenous Mbd4 1/6–8 transcript, translation initiation from the Kozak sequence in exon 1 leads to premature termination. However, an alternative Kozak sequence in exon 6 could potentially direct translation of a peptide through an open reading frame in exons 6–8. CH12.F3 cells were stably transduced with empty or MBD4 exon 6–8 constructs, using the Kozak sequence in exon 6, to generate independent stable pools and analyzed in Western assays. The endogenous expression profile for full length MBD4 (68 kD) is preferentially nuclear (fig. 1B, 4D), however the truncated MBD4 6–8 species (12 kD) can be found in both the cytoplasm and the nucleus in 3 independent experiments (fig. 4D). The truncated peptide will have lost a NLS predicted to be in exon 3 but retains the NES predicted to be in exon 7 (fig. 1A, 4A). The short length of this peptide in combination with the loss of the NLS may allow diffusion from the cytoplasm into the nucleus. These findings raise the possibility that Mbd4 ablation is incomplete in Mbd4^Δ2−5/Δ2−5^ mice and that a truncated form of MBD4 is expressed at low levels. However, efforts to detect the putative truncated form of MBD4 in Mbd4^Δ2−5/Δ2−5^ B cells activated to undergo CSR by Western analyses were inconclusive (data not shown). This could be due to reduced expression that is below the level of detection with our assays or because the truncated protein is not expressed. We cannot distinguish between these possibilities.

### Two models to account for CSR function in Mbd4^Δ2−5/Δ2−5^ B cells

We consider two hypotheses to reconcile reduced and unimpaired CSR function in Msh2^+/−^ and Mbd4 deficient B cells, respectively. It is possible that full length MBD4 is a negative modulator of MMR proteins and this function is abolished in Mbd4 deficient B cells. In this scenario, we predict that over-expression of MBD4 would reduce CSR as MMR protein levels are further sequestered by MBD4. To test this possibility we transduced full length Mbd4, flipped Mbd4 (to control for construct length) and empty vector in normal splenic B cells activated with LPS+IL4. Transduction was successful as evidenced by GFP+ B cells detected by FACS and these cells underwent CSR to IgG1 (fig. S1). However, the frequency of GFP+ B cells obtained following transduction with full length Mbd4 was severely reduced compared to controls. This implies that MBD4 overexpression causes cellular toxicity making any interpretation difficult.

An alternative model predicts that MBD4 is a positive modulator of CSR and/or that a truncated version of MBD4 expressed in Mbd4^Δ2−5/Δ2−5^ B cells preserves this function. To examine this possibility, we ectopically expressed truncated MBD4 (exons 6–8) in WT CH12.F3 cells and found no effect on CSR (unpublished data, F.G., A.L.K.). This may have occurred because we could not discern an increase of CSR activity above WT levels or because there is no function for the truncated protein. To further examine this issue, we undertook knockdown of Mbd4 using newly designed or previously validated shRNAs [28] in CH12.F3 cells. Although CSR was impaired in some of these cultures, MBD4 protein expression was not reduced, indicating off target effects and rendering the interpretation of these studies complex (data not shown). Thus, the function of a putative truncated MBD4 in Mbd4^Δ2−5/Δ2−5^ B cells remains unresolved.

## Discussion

We show that MBD4 is up-regulated in activated splenic B cells consistent with an involvement in CSR. However, Mbd4^Δ2−5/Δ2−5^ B cells, which fail to express full length MBD4 are capable of WT levels of CSR. This was surprising because Mbd4 deficiency is also associated with reduced levels of MMR proteins MSH2, MLH1 and PMS2, which are required for CSR. We show that Msh2 is haploinsufficient for CSR. Furthermore, we found that MSH2 levels in Mbd4 deficient B cells are lower than in Msh2^+/−^ B cells, which are impaired for CSR. Why are the low levels of MSH2 not limiting for CSR in Mbd4 deficient B cells? Our results suggest that MSH2 per se is not required for CSR, contrary to previous conclusions [25] and this finding has important implications for our thinking regarding the mechanism of CSR.

Our studies reveal that in Mbd4^Δ2−5/Δ2−5^ B cells aberrant Mbd4 transcripts, originating from the 3′ end of the locus, could potentially encode a truncated MBD4 peptide which functions to support CSR either directly or indirectly. It is unlikely that the putative truncated MBD4 acts via a glycosylase function, since a similar 6–8 MBD4 construct expressed in *E. coli* is devoid of thymine glycosylase activity [6]. It is possible that a critical scaffolding function with MMR proteins is retained in truncated MBD4 6–8, since this function was mapped to the MBD4 C-terminus [7]. An alternative possibility is that the two lncRNAs encoded in part at the 3′ end of the Mbd4 locus and retained in Mbd4^Δ2−5/Δ2−5^ B cells are critical for CSR. Finally, it is possible that the truncated MBD4 is involved in interacting with- and stabilizing other MMR proteins. Although we cannot currently distinguish among these alternatives, our studies imply that the 3′ end of the Mbd4 locus encodes information required for CSR function.

We considered two models to account for the paradoxical CSR phenotypes in Msh2^+/−^ and Mbd4 deficient B cells. The first is that full length MBD4 is a negative modulator of MMR proteins and this function is abolished in Mbd4 deficient B cells. Due to technical limitations we were unable to confirm or refute this possibility. Nevertheless, this model does not accommodate the observation that CSR is impaired in Msh2^+/−^ B cells where both MSH2 and MBD4 protein concentrations are reduced. The second model predicts that MBD4 is a positive modulator of CSR. Our studies raise the possibility that residual MBD4 protein expression in Mbd4^Δ2−5/Δ2−5^ B cells is sufficient to enable CSR. Although, we found no increase of CSR frequency when the truncated protein spanning exons 6–8 was ectopically expressed in CH12.F3 cells, this may reflect our inability to discern additional switching above the already high level of CSR in these cells. Future experiments directed toward targeted deletion of the 3′ end of the Mbd4 locus and ablation of all Mbd4 expression will be required to clarify this issue in murine B cells.

## Materials and Methods

### Mice, B cell culture and FACS analysis

Mice were purchased from Jackson Laboratories or maintained in colonies at the University of Illinois College of Medicine under pathogen-free conditions. All procedures involving mice were approved by the Institutional Animal Care Committee of the University of Illinois College of Medicine (Protocol # 11-189). All efforts were taken to minimize animal suffering. Mbd4^Δ2−5/+^ mice were backcrossed more than nine times to the C57BL/6 strain [6] and were bred to obtain WT and KO littermates. Splenocytes from Msh2 [29] or Mlh1 [30] deficient mice backcrossed to the C57BL/6 strain were kindly provided by Dr. Stavnezer (University of Massachusetts Medical School, MA). CD43- B cells were isolated, cultured and analyzed by FACS as previously described [2].

### CH12.F3 cell culture and FACS analysis

CH12.F3 was cultured in RPMI supplemented with 6% fetal calf serum, 4 mM glutamine, 50 µM β-mercaptoethanol, and pen/strep. at a cell density of 1.25×10^5^ cells/ml. Cells were stimulated with IL4 (5 ng/ml) (R & D Minneapolis, MN), TGF-β (0.2 ng/ml) (R & D Minneapolis, MN), and CD40L (25% vol/vol), (CIT), for 24 or 40 hours, whereupon cells were analyzed for surface IgA by flow cytometry using biotinylated rat anti-mouse IgA (0.5 mg/ml) (Southern Biotech) was used in combination with streptavidin conjugated APC (0.2 mg/ml) (eBioscience 17-4317-82).

### Quantitative (q) RT-PCR

RNA was isolated from B cells (2×10^6^) that were activated for 24 or 48 hours with LPS+IL4 activation using Trizol (Invitrogen) according to the manufacturers instructions. Q-RT-PCR were performed as described [2] except that samples were normalized using 18S rRNA [31]. QRT-PCR was performed using SYBR^©^ Green (ABI) and the ABI 7600 FAST SYSTEM in combination with the primers for 18S RNA [31], γ1 and γ3 GLT [2], Mbd4-F5, 5′-ACGATCTCCTGTCAAAAACC-3′, and Mbd4-R1, 5′-GCCCTTAGAAACCGAAGCACA-3′, Msh2-F1, 5′-GAGTCGGGGCTGGTGACAGT-3′, Msh2-R1 5′-AATGGGTGGCAAACATGCAA-3′; Mlh1-F1, 5′-ATCAGCCCTCAGAACTGTGA-3′, and Mlh1-R1, 5′-CCCTGTCGTGGGTCTAGCTG-3′. Mbd4-F1, 5′-AGGGATGGAGAGCCCAAAC-3′ was paired with Mbd4-R1 in RT-PCR (fig. 4B). CN781668-F3, 5′-AGTGACAGGCTGAGGCTACC-3′, together with, CN781668-R3, 5′-TTTCAGTTTGTTAGAAGCCAGTG-3′, or AID-FWD1, 5′-CCATTTCAAAAATGTCCGCT-3′, and AID-REV1, 5′-CAGGTGACGCGGTAACACC-3′ [2], were also used (fig. 4B). For q-PCR standard curves were generated for each primer pair using cDNA from B cells activated with LPS+IL4, or LPS alone, for 48 hours.

### Immunoprecipitation and Western assays

Cytoplasmic and nuclear extracts were prepared as described [32] with modifications. For IP assays, DTT was omitted from nuclear extracts and final NaCl was 100 mM. IPs on nuclear extracts (100 µg) were pre-cleared with protein-A beads (60 µl) (Pierce) and anti-GFP (1 µl of 1:5 dilution) (Abcam, 290) then immunoprecipitated with anti-MLH1 (Abcam, 92312), anti-MBD4 (Abcam, 12187), or anti-GFP (1 µg) Abs and incubated at 4°C/2h. Protein-A beads (30 µl) were added and incubated at 4°C/1h then washed and boiled in SDS loading buffer. Proteins were resolved by SDS-PAGE and electrotransfered to Immobilon-P® PVDF membranes (Millipore, IPVH00010) per the manufacturers instructions. Membranes were blocked in 5% milk in TBS with 0.2% Tween (TBS-T) then probed in 2% milk with TBS-T. For Western blots, membranes were probed with anti-MLH1 (1∶10,000), anti-MSH2 (1∶400; Santa Cruz, sc-494), anti-PMS2 (1∶5000; BD Pharmingen, 556415), anti-MBD4 (1∶4000), and anti-β-ACTIN (1∶25,000, Sigma, A8316), washed and incubated with horseradish peroxidase (HRP) linked secondary Abs (1∶10,000; Amersham) then visualized using a chemiluminescent substrate (Pierce, 34080) and films (Denville Scientific, E3012).

### MBD4 ectopic expression and CH12.F3 transduction

MBD4 exon 6–8 cDNA was PCR amplified using Mbd4-6-8-FWD, 5′-AGATCTAAGATGGCCATCCCTGTGCTGTGGGAGTTTCT-3′, and 1/6-8-REV, 5′-TCAAGATAGACTTAATTTTTCATGATTCTCCC-3′, cloned into TOPO-TA (Invitrogen), and subcloned into pMSCV-puro (Clontech). Retrovirus was made by transfecting MBD4 1-8, MBD4 1-8-flipped, MBD4 6-8 and empty pMSCV constructs (4 µg), and a plasmid expressing the VSV-gp glycoprotein (1 µg) into Platinum GP cells (Cell Biolabs). Cells were infected by spinoculation then cultured in puromycin (2 ng/ml) (Sigma) for 2–5 days. When cells reached a viable density (1×10^5^ cells/ml), 1–1.5×10^7^ cells were taken for Western analyses.

### Prediction of NLS and NES

Prediction of the NLS in MBD4 was carried out as described in http://www.sbc.su.se/~maccallr/nucpred/ [33]. The NES in MBD4 was predicted using the NetNES 1.1 Server as described in http://www.cbs.dtu.dk/services/NetNES/ [34].

## Supporting Information

Figure S1 **Ectopic MBD4 expression in WT splenic B cells is associated with cellular toxicity.**
(PDF)
