# Supplementary material for: Complex Relationship between Mismatch Repair Proteins and MBD4 during Immunoglobulin Class Switch Recombination
Source: PLoS One. 2013 Oct 29;8(10):e78370. doi: 10.1371/journal.pone.0078370 (PMC3812156; doi:10.1371/journal.pone.0078370)
Supplement: Figure S1 — Ectopic MBD4 expression in WT splenic B cells is associated with cellular toxicity. (PDF) [file pone.0078370.s001.pdf]

Complex relationship between mismatch repair proteins and Mbd4 during immunoglobulin class switch recombination

Fernando Grigera, Alfonso Bellacosa, and Amy L. Kenter

SUPPLEMENTARY INFORMATION

Supplementary Figure S1

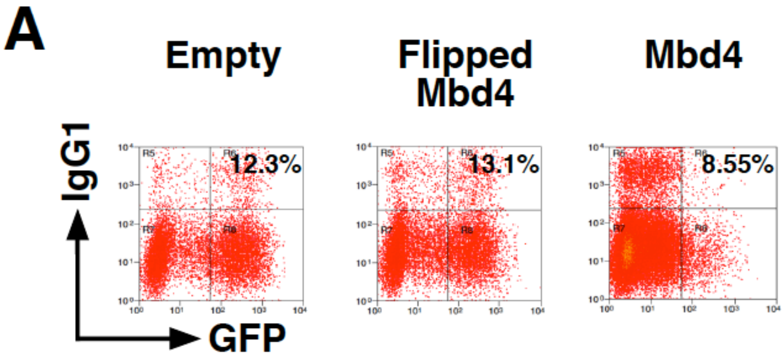

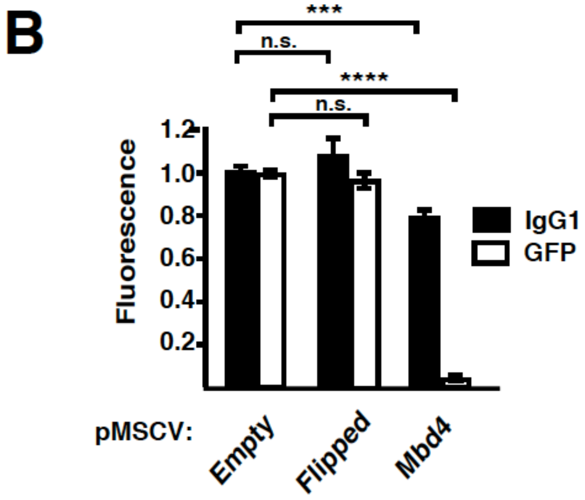

Suppl. Figure S1. Ectopic MBD4 expression in WT splenic B cells is associated with cellular toxicity. B cells were activated with LPS+IL4 for 24 hours, transduced with virus from pMSCV that was empty, or expressed Mbd4, or Mbd4 flipped. Cells were cultured for an additional 48 hours, and then monitored for IgG1 switching. **A)** Representative two color FACS analysis of anti-IgG1 (APC) versus GFP. The population of viable cells was determined by forward and side scatter. Gates are drawn to display the percent of GFP positive cells (green). The percent of IgG1 switched cells within the GFP positive population is shown in the upper right hand quadrant of each histogram. **B)** Summary of transduction experiments normalized to Empty vector and set to 1.

Averages from four independent transductions with SEM are shown. P values were calculated using Student's two tailed *t* test; $p < 0.05$ (*), $p < 0.01$ (**), $p < 0.001$ (***), $p < 0.0001$ (****).
